# The Small Breathing Amplitude at the Upper Lobes Favors the Attraction of Polymorphonuclear Neutrophils to *Mycobacterium tuberculosis* Lesions and Helps to Understand the Evolution toward Active Disease in An Individual-Based Model

**DOI:** 10.3389/fmicb.2016.00354

**Published:** 2016-03-29

**Authors:** Pere-Joan Cardona, Clara Prats

**Affiliations:** ^1^Unitat de Tuberculosi Experimental, Fundació Institut d'Investigació en Ciències de la Salut Germans Trias i Pujol, Universitat Autònoma de Barcelona, CIBERESBadalona, Spain; ^2^Escola Superior d'Agricultura de Barcelona, Departament de Física, Universitat Politècnica de Catalunya – BarcelonaTechCastelldefels, Spain

**Keywords:** NetLogo, Individual-Based Model, tuberculosis, polymorphonuclear neutrophils, Th17, bacillary drainage, breathing amplitude, alveolar macrophages tolerability

## Abstract

Infection with *Mycobacterium tuberculosis* (*Mtb*) can induce two kinds of lesions, namely proliferative and exudative. The former are based on the presence of macrophages with controlled induction of intragranulomatous necrosis, and are even able to stop its physical progression, thus avoiding the induction of active tuberculosis (TB). In contrast, the most significant characteristic of exudative lesions is their massive infiltration with polymorphonuclear neutrophils (PMNs), which favor enlargement of the lesions and extracellular growth of the bacilli. We have built an individual-based model (IBM) (known as “TBPATCH”) using the NetLogo interface to better understand the progression from *Mtb* infection to TB. We have tested four main factors previously identified as being able to favor the infiltration of *Mtb*-infected lesions with PMNs, namely the tolerability of infected macrophages to the bacillary load; the capacity to modulate the Th17 response; the breathing amplitude (BAM) (large or small in the lower and upper lobes respectively), which influences bacillary drainage at the alveoli; and the encapsulation of *Mtb*-infected lesions by the interlobular septae that structure the pulmonary parenchyma into secondary lobes. Overall, although all the factors analyzed play some role, the small BAM is the major factor determining whether *Mtb*-infected lesions become exudative, and thus induce TB, thereby helping to understand why this usually takes place in the upper lobes. This information will be very useful for the design of future prophylactic and therapeutic approaches against TB.

## Introduction

Tuberculosis is still a major threat to humankind, causing up to 1.5 million deaths so far (World Health Organization, 2015). *Mycobacterium tuberculosis* (*Mtb*) is able to reach the alveolar spaces via micrometric aerosols. Once it has reached this site, it is phagocytosed by alveolar macrophages (AMs) and can grow intracellularly until it causes apoptosis or necrosis, thereby becoming extracellular (Lee et al., 2006). These bacilli are drained toward other alveolar spaces, where they cause its infection, or again infect the AMs that come from the interstitial space to replace those that have disappeared (Hashimoto et al., 2013; Hussell and Bell, 2014). This takes place until a certain number of AMs have been infected and destroyed, which induces the local inflammatory response that allows sufficient bacilli to reach the lymphatic nodes and trigger the immune response. This response is mainly based on the proliferation of specific T CD4 lymphocytes that are able to activate infected AMs by secretion of IFN-γ (North and Jung, 2004; Vilaplana et al., 2014). The majority of subjects subsequently control the infection, thus resulting in minimal and well-controlled lesions. However, in a few cases, the infection is able to develop large progressive lesions, thereby causing a relevant destruction of the lung parenchyma that impairs their functionality and generates active disease. Data from necropsies of early TB cases performed in the pre-antibiotic era (Cardona, 2015) show how progressive lesions are mainly built by infiltration of the parenchyma by polymorphonuclear neutrophils (PMNs), intragranulomatous necrosis and liquefaction in the presence of a high bacillary load. In contrast, lesions built with AMs and epithelioid cells appear to have a controlled size and a lower bacillary load.

Data from *in vivo* experimental modeling in either mice (Marzo et al., 2014), rabbits (Converse et al., 1996), goats (de Val Pérez et al., 2011), or monkeys (Flynn et al., 2015) also show the same pattern. Thus, *in vitro* experimental data demonstrate that the ability to attract PMNs is related to the tolerability of the infected AMs to a certain bacillary load, thus causing either apoptosis or necrosis (Lee et al., 2006). In this context, apoptosis would cause a limited intracellular growth in the alveolar AMs and a limited inflammatory response due to a lack of PMNs, whereas higher tolerability or an inability to cause apoptosis allows higher intracellular bacilli concentrations, which is related to a marked neutrophilic infiltration (Gan et al., 2008).

Equally, the quality of the immune response is also responsible for further infiltration. It is known that the induction of a Th17 response favors the attraction of PMNs to the lesions once specific lymphocytes reach them and begin to secrete IL-17. This Th17 response is triggered against extracellular pathogens as a result of the cytokine profile generated by their presence (Korn et al., 2009).

Two major factors are also related to the progression toward active TB. First of all, it appears that the upper lobes accumulate by far the largest number of TB lesions (Dock, 1954). This has been related to the mechanics of the respiration process, which is influenced by the force of gravity, thereby impairing the breathing amplitude (BAM) in the upper lobes while enhancing it in the lower regions thanks to the force generated by the diaphragm. This small amplitude in the upper lobes (Guo et al., 2011) could allow the local accumulation of bacilli, thereby attracting PMNs.

Another factor that helps control lesion progression is the encapsulation process that takes place in large mammals (Peake and Pinkerton, 2015). In these animals there is a need to structure the parenchyma into a net of septae that connect to the visceral pleura, thus facilitating the ventilation process without disrupting the fragile lung parenchyma (Peake and Pinkerton, 2015). This net structures the parenchyma into cubes with a volume of around 1 cm^3^, known as secondary lobes, containing four acini, each of which has four alveolar sacs containing four alveoli (Webb, 2006). The fibroblasts that maintain these septae can react quickly to the presence of any minimal lesion in the parenchyma by encapsulating it and stopping its progression by fibrosis and calcification (Gil et al., 2010).

We have built an individual-based-model (IBM) to understand how these four factors can influence the progression toward active TB and have uncovered the key role played by drainage activity, which can be compensated by the macrophage tolerability and by changing the quality of the immune response to an anti-Th17 one. The encapsulation process also plays a crucial role, although it also depends on the speed of lesion growth, which can overcome encapsulation when intense enough.

## Materials and methods

The model is described following the IBM standardized protocol ODD (Overview, Design concepts, and Details) (Grimm et al., 2006, 2010). This protocol consists in formalizing the IBM with three blocks, which are subdivided into seven optional subcategories, namely Purpose, Entities, State variables and scales, Process overview and scheduling, Design concepts, Initialization, Input data, and Submodels.

### Overview

#### Purpose

The objective of this IBM is to analyse and understand the influence of four factors, namely the tolerability of the infected AM (BLTOL), the anti-Th17 response, bacillary drainage (BAM) and the encapsulation process, on the progression toward active TB.

#### Entities, state variables, and scales

The fundamental entities in this model are alveoli and bacilli. The IBM has been built using the NetLogo structure (Tissue and Wilensky, 2004) based on *patches* (spatial cells) and *turtles* (individuals). Thus, patches represent individual alveoli and turtles represent individual bacilli. The time step is 1 h, which is small enough to allow the modeling of the bacilli growth cycle (around 24 h) and wide enough to allow computationally feasible simulations. The simulated period is 42 days from initial infection (7 weeks), as the acute phase we are aiming to reproduce is observed at 3–4 weeks post-infection and the lesions are stabilized at around 6 weeks post-infection (Cardona et al., 2003).

##### Patches or spatial cells

A 2D grid of 46 × 46 alveoli is defined, each alveolus being represented by a spatial cell or patch (in humans, 0.3 mm diameter) (Suarez et al., 2012). The whole grid is equivalent to a secondary lobule as the average diameter of the secondary lobule is from 10 to 25 mm of diameter (Webb, 2006). This 2D grid is enough to represent an ensemble of connected alveoli, since the model does not include the lung 3D structure at this stage.

Each patch initially contains a single alveolar macrophage (AM) (Suarez et al., 2012; Peake and Pinkerton, 2015) whose state is determined by the evolution of the infection. Patch variables are mostly related with the characteristics of the AM and other cells that they contain, each state being represented by a specific color as follows:

- White patch: alveolus with the single initial AM;
- Black patch: alveolus whose AM has been destroyed by the bacilli;
- Blue patch: alveolus whose initial AM has been replaced by another one from the interstitium after its destruction, i.e., without the need for extravasation caused by an inflammatory response to enter the cell;
- Violet patch: alveolus with one activated AM (aAM);
- Pink patch: alveolus that has been occupied by PMNs (neutrophilic infiltration); and
- Dark pink patch: necrotized alveolus (alveolus with caseum).

In addition, a brown patch defines the presence of interlobar fibroblasts. Initially they are found in interlobar septae, but they are able to encapsulate lesions under specific conditions (Gil et al., 2010).

All these cells (AMs, PMNs, fibroblasts) do not necessarily occupy the entire available space in the alveolus, but their presence and characteristics are shown through the patch color. In fact, while the mean diameter of human alveoli is 0.3 mm, AMs size around 20 microns diameter and PMNs size around 8 microns diameter (Krombach et al., 1997). The interstitial space that provides new interstitial AM and the capillary net responsible for the entrance of AM and PMNs once the inflammatory response is triggered are not explicitly modeled because their size is negligible in front of alveolar space.

##### Turtles or individuals

Bacilli are the agents (individuals) and their properties are related to their state within the alveolar growth cycle, which is also identified with colors: initial bacillary load (Ibac, blue), bacilli growing intracellularly (Ibac, red), non-growing extracellular bacilli (Ebac, in green), growing Ebac (brown), dormant bacilli (Dbac, in orange) or killed bacilli (Kbac, in yellow). When growing, the time within the growth cycle (reproduction time) is also controlled.

##### Global variables

The global variables are mostly related to the bacillary load of the system, as well as the PMN and aAM concentration and spatial distribution. A further important global variable is related to activation of the immune response, which can acquire values of 0 (no immune response yet) or 1 (immune response already activated).

#### Process overview and scheduling

As mentioned above, the model was constructed in NetLogo (Tissue and Wilensky, 2004), which is well suited for modeling individual-based systems in a user-friendly interface. The user is allowed to select different conditionings and properties of the system to be simulated, using a set of sliders (see Details sub-section).

The simulation starts with definition of the initial configuration, in which a certain number of intracellular bacilli are randomly distributed in the space. The initial bacillary load can be chosen by the user. All alveoli are set to the initial single AM state (white). If encapsulation is considered, the limits of the space are changed to the encapsulated state (brown). The model assumes discrete time steps of 1 h, as mentioned. Each hour, all individuals execute a series of actions, and their variables are updated immediately. The characteristics of the patches are also immediately updated as a consequence of evolution of the infection. Patches (i.e., alveoli) are submitted to actions that may affect the bacilli they contain.

**Figure 1:**
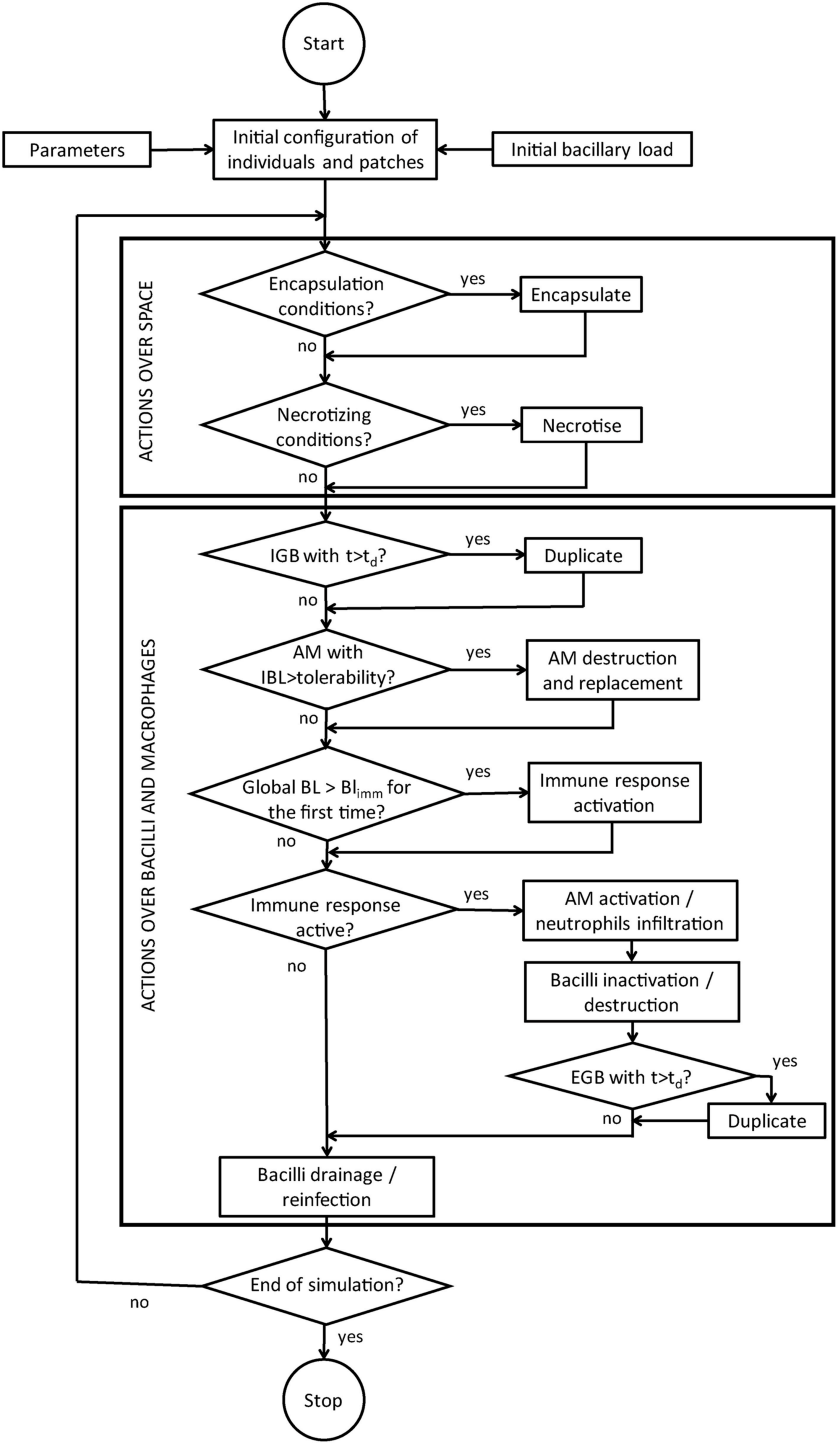
**Flow chart of the computer code**.

The actions for individuals are, when possible: reproduce (either intracellularly or extracellularly), become extracellular when the macrophage is destroyed, be drained, be killed by the immune response, and re-infect. The actions for patches can be distinguished between those that affect the space and those that affect the AM contained in patches. The former include get encapsulated and get necrotized, whereas the latter (actions concerning the AM) are, when appropriate, be destroyed by intracellular bacilli growth, be replaced after destruction, and be activated by inflammatory response. Finally, when the global bacillary load exceeds a certain threshold, the immune response is activated everywhere. Global variables are updated at the end of each time step. Figure 1 shows the flow diagram for the computational model.

### Design concepts

#### Basic principles

The model is based on the observations and theories referred to in the introduction to this paper, which are summarized in a recent review (Cardona, 2015).

#### Emergence

Emerging phenomena are mainly related to the global dynamics of the infection. In particular, the spatial patterns that emerge from simulations are especially interesting as they are a reflection of the different kinds of lesions that may be found in a TB-infected lung (proliferative and exudative), and are the result of non-trivial interactions between the processes considered.

#### Interaction

Local interactions are mainly driven by space. Thus, intracellular growth, for example, is limited by the bacillary load tolerability (BLTOL) of the AM, whereas the properties of neighboring patches (encapsulation, necrosis) condition the evolution of a single patch. Interactions at a global level are related to activation of the immune response, which affects the whole system. In addition, global interactions are also given by the possibility of the bacilli being drained and reinfecting a different alveolus.

#### Stochasticity

Randomness is introduced at all levels of the simulation. Some actions are associated with a certain probability and are thus executed according to a stochastic number. All parameters are affected by Gaussian noise.

#### Observation

Output data show the global evolution of each class of bacilli (intracellular, extracellular, dormant and killed) as well as the amount of AM in each state. In addition, the evolution of PMNs, AM and necrosis are also reported. Data are exported to an external data file. Moreover, the graphical interface continuously shows the spatial evolution, thus allowing the observer to see the different patterns of lesions.

### Details

#### Initialization

**Figure 2:**
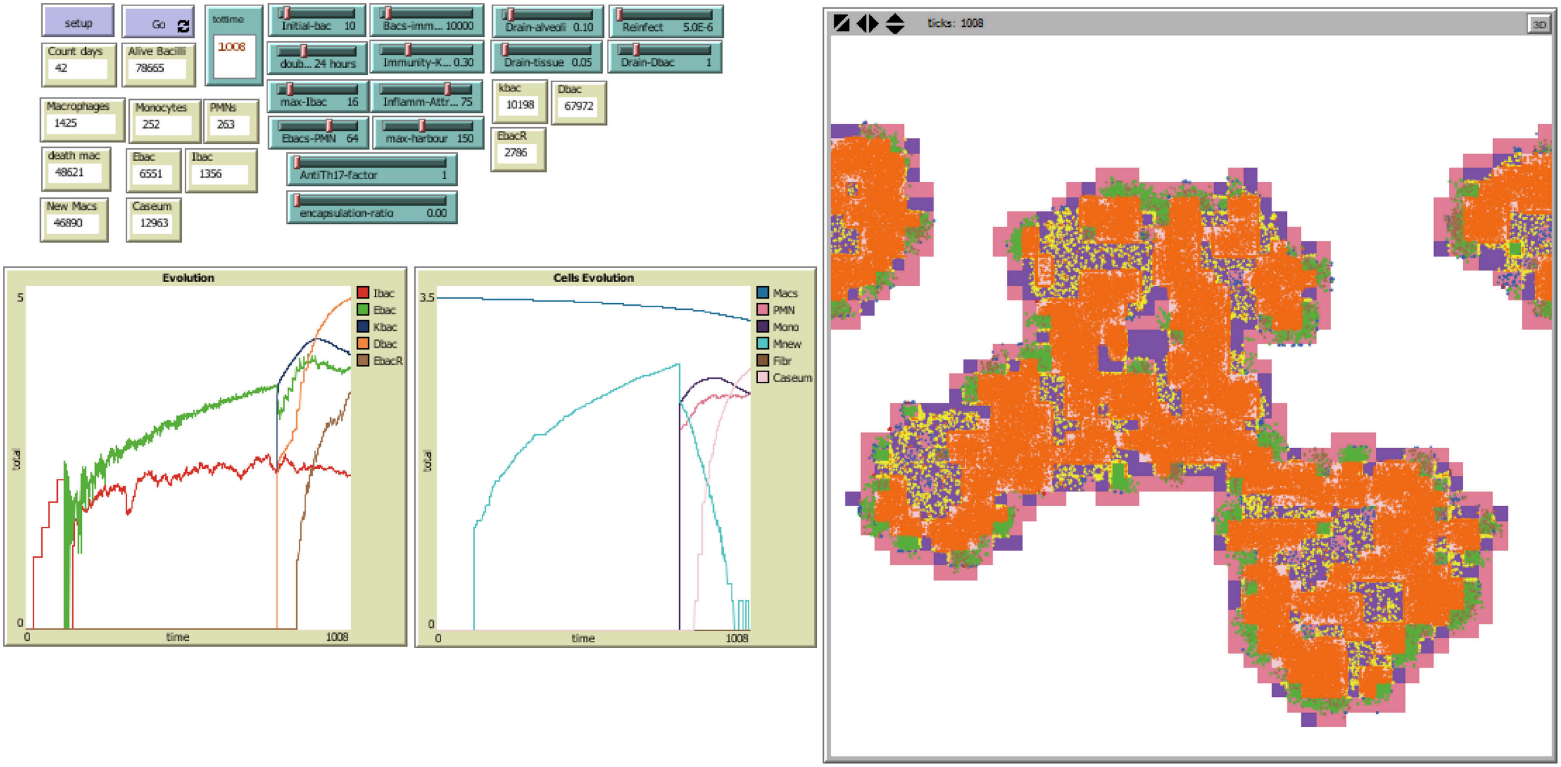
**NetLogo's user interface, with model TBPATCH**. Green sliders are the parameters that can be changed by the user. Brown boxes show the evolution of different variables during a simulation. The left window is the world representation, where spatial evolution of the infection can be seen. ***Colors:*** Alveoli: **White**: when a single AM is present; **Black**: when the AM is destroyed; **Blue**: when the AM is destroyed and replaced by another AM from the interstitium; **Violet**: when an activated AM (aAM) is present; **Pink**: when PMNs occupy the alveoli; **Brown**: capsule. Bacilli: **Blue** challenge bacilli; **Red**: Intracellular bacilli (Ibac); **Green**: Extracellular (Ebac); **Brown**: growing Ebac; **Orange**: dormant (Dbac); **Yellow**: killed bacilli (Kbac).

The following sliders can be used to select the characteristics and initial conditions of the system to be simulated, as shown in Figure 2.

*Initial-bac:* used to define the initial bacillary load of the challenge. Each initial bacillus will be located randomly.
*Doubling-time:* refers to the doubling-time of the bacilli.
*Max-Ibac:* this is the maximum bacillary load tolerated by the AM. Once reached, the cell is destroyed. This parameter defines the bacillary load tolerability (BLTOL).
*Ebacs-PMN:* this is the bacillary load required to attract infiltration of the alveoli by PMNs.
*Bacs-immunity:* the total bacillary load in the grid required to trigger the immune response.
*Immunity-Kbac:* chance of killing intracellular bacilli once an AM is activated.
*Inflamm-attract:* global number of bacilli that should be around an infected AM to be activated once the immune response has been triggered.
*Max-harbor:* maximum bacillary load that can be supported by an alveolus. Once this number is reached, the induced inflammatory response has attracted sufficient cells (mainly PMNs) that it is finally destroyed, thereby inducing necrosis, and all the immerged bacilli become dormant (Dbac).
*Drain-alveoli:* maximum distance that can be reached by Ebac or Dbac every time-point, representing the BAM.
*Drain-tissue:* the drainage capacity of Ebac or Dbac once they are located in PMN-filled alveoli, thus representing the BAM. We have considered this to be equal to Drain-alveoli/2 in all cases.
*Reinfect:* this is the probability of distant drainage of Dbac, thus representing the process observed once immune response is reached by those old aAMs that become foamy macrophages (FMs). These FMs drain out of the lesions (plenty with Dbac) and some of them destroyed on the way to reaching the upper bronchi.
*Drain-Dbac:* this is the distance to which Dbac are drained as a result of the reinfection phenomenon.
*Anti-Th17 factor:* to reduce the effect of the Th17 response, which favors the entry of PMNs into the different lesions once the immune response is reached.
*Encapsulation-ratio:* this defines the ability to encapsulate, although we have defined only two conditions: when no encapsulation is considered, the capsule frame is eliminated from the program. It is only informed.

#### Sub-models

##### Encapsulation

This sub-model affects spatial cells and is only applied when the *encapsulation-ratio* is set to 1 in the initial conditions. The encapsulation of a certain patch requires two simultaneous conditions: (i) at least one neighboring cell must be already fibrosed (brown); and (ii) at least one neighboring cell has a non-resident AM (sky), an activated AM (aAM) (violet), PMN infiltration or necrosis (pink). If these conditions are satisfied, encapsulation takes place.

It is possible to set an *Encapsulation-ratio* lower than 1. In this case, encapsulation would take place at this ratio.

##### Necrosis

This sub-model also affects spatial cells. The conditions for a patch to get necrotized are, indistinctly: (i) a neighboring cell already has necrosis; or (ii) the local bacillary load exceeds *Max-harbor*. If one of these conditions is satisfied, the patch is necrotized.

##### Bacillary growth

The individual reproduction-time of the bacilli is updated after each time step. Ibac doubles once the duplication time is achieved (*Doubling-time*), giving rise to the appearance of two intracellular bacilli with age zero.

In the case of Ebac, the increase in reproduction-time and duplication only occurs when the local patch is neutrophilic. A maximum bacillary load is defined for each patch (*Max-harbor*). Once this is reached, no more bacillary growth is accepted and the whole bulk becomes dormant.

##### Infected macrophage destruction

A degree of tolerability of AM to a maximum intracellular bacillary load is initially defined (*Max-Ibac* or BLTOL). Once this concentration is reached, the AM is destroyed. Local bacilli subsequently become extracellular (Ebac).

##### Immune response activation

When the total bacillary load exceeds the threshold given by *Bacs-immunity*, the immune response is immediately activated. This status is maintained until the end of the simulation.

##### Destroyed AM replacement

The replacement of a destroyed macrophage depends on the immune status of the system.

If the immune response has not yet been activated, the destroyed macrophage may be replaced by a new AM as long as the bacillary load required to attract infiltration of the alveoli by PMNs (*Ebacs-PMN*) is not exceeded. This AM is assumed to enter the alveolus through the interstitial space, which is not explicitly represented in the model because its size is negligible. Otherwise, if the total bacterial load of neighboring patches is greater than the ratio *Bacs-immunity*/Inflamm-attract, the alveolus undergoes neutrophilic infiltration (see below).

Once the immune response has been activated, and if the total bacterial load of neighboring patches is lower than the ratio *Bacs-immunity/Inflamm-attract*, the AM is replaced by a new (non-activated) one. If this ratio is exceeded but *Max-harbor* has not yet been reached, AM activation or neutrophilic infiltration takes place, depending on the corresponding sub-model.

##### AM activation and neutrophilic infiltration

This sub-model takes into account the need for a certain bacillary load or infected alveoli in the surroundings of the patch considered in order to attract the PMNs or activate an AM into an aAM. It is applied to those alveoli with a non-activated macrophage (white and sky patches) and to those alveoli with a destroyed AM (black), whenever the total bacterial load of neighboring patches is higher than the ratio *Bacs-immunity/Inflamm-attract*, the *Max-harbor* is not surpassed and once the immune response has been activated. Then, the probability of suffering a neutrophilic infiltration is given by a new indicator: the local percentage of Ebac over Ebac and Ibac, modulated by the factor *Anti-Th17*. The neutrophilic infiltration takes place with this probability and through the capillary net, which is not explicitly represented in the model because its size is negligible. Alternatively, the AM is activated (in white and blue patches) or a new aAM replaces the destroyed one (in black patches).

##### Bacillary inactivation and destruction

The inactivation or destruction of Ibac and Ebac requires the immune response to be present. As such, this sub-model only applies once the immune response has been triggered.

In general, the destruction and inactivation of these bacilli may only occur when an activated AM (aAM) is present (i.e., in violet patches). Destruction therefore takes place with a probability *immunity-Kbac*, and inactivation into Dbac takes place with a probability of *immunity-Kbac/10*.

The inactivation of Ebac into Dbac in an alveolus in which the macrophage has been destroyed by bacillary growth is also possible (i.e., without the presence of an aAM). This occurs with a probability of *immunity-Kbac/10*.

##### Breathing amplitude (BAM) for bacillary drainage and reinfection

There are two mechanisms for drainage: via a foamy infected macrophage (FM) and via the tissue. The distance at which bacilli can be drained is randomly chosen between 0 (no drainage) and *drain-Dbac* (FM-driven) or *drain-tissue* (tissue-driven).

FM-driven drainage results in an endogenous reinfection process. This occurs for all Dbac with a probability of *Reinfect*. These bacilli may also be killed during this drainage, with a 10-fold increase in this probability.

Tissue-driven drainage is considered for all Dbac in neutrophilic infiltration and is a consequence of the BAM. In both cases, those Dbac that move into an alveolus with an AM become intracellular (Ibac), whereas those that move into a neutrophilic infiltration become extracellular (Ebac).

Tissue-driven drainage is also considered for Ebac in the following cases:

- When they are released from a destroyed AM.
- When they are in a neutrophilic infiltration.
- When an Ebac replicates, both bacilli may be drained via tissue.

Those Ebac that move into an alveolus with an AM return to the intracellular state.

### Parameterization and simulation scheduling

The local sensitivity analysis and parameter estimation followed an iterative process that entailed successive simulations in order to (i) delimit the ranges where parameters and outcome results made biological sense, and (ii) identify those parameters that were more sensitive with regards to different outcome variables. During this stage, the parameters were varied on-at-a-time a certain percentage (except for specific parameters like encapsulation, that we only accounted for on/off) and their effect was firstly checked on lesion patterns observed in a qualitative way and secondly checked on the outcome variables that we finally selected as indicators. This involved several rounds from which we can highlight an initial broad analysis where parameters were varied at wide ranges (typically 100 or 50%), a subsequent stage for estimating parameters taking into account what we observed at the first stage, and a final sensitivity analysis where parameters were varied smaller percentages (typically 10%) and from which we finally selected the four parameters to be studied, taking into account the basic principles of the model.

This process allowed the selection of those values or ranges that provided biologically viable results (Table S1). The aim was to observe the different patterns in simulations that represented around 50 days post-infection. As such, an initial bacterial load of 10 bacilli was chosen, with a duplication time of 24 h. The threshold for activation of the immune response was set to 10^4^ bacilli, a value similar to that determined by Vilaplana et al. (2014). Other non-measurable parameters were estimated to provide a visible but non-exaggerated effect. In particular, *Immunity-Kbac* was set to 30%, *Inflamm-Attract* was set to 75 bacilli, *Max-harbor* was set to 150 bacilli*, Reinfect* was set to 5 × 10^−4^%, *Drain-Dbac* was set to 1 patch h^−1^ (equivalent to 0.3 mm h^−1^), and *Ebacs-PMN* was set to 64 bacilli. The parameter space exploration also reported the relationship *Drain-tissue* = *0.5* × *Drain-alveoli*.

Once these 7 parameters were fixed, a set of *in silico* experiments was designed in order to study the effect of the 4 processes and factors considered. The selected values to explore were as follows:

- *Max-Ibac or BLTOL:* 16, 32, and 64 bacilli, as obtained from experimental data (Lee et al., 2006).
- *Drain-alveoli or BAM:* 0.1, 0.2, and 0.3 patch h^−1^ (equivalent to maximum velocities of 0.03, 0.06, and 0.09 mm h^−1^, respectively).
- AntiTh17-factor: 1 and 10.
- *Encapsulation*: 0 (no encapsulation) and 1.

The simulations were scheduled using the Behavior Space tool of NetLogo. All values were combined, i.e., a total of 36 possible combinations were explored (i.e., a grid-search of 3 × 3 × 2 × 2 combinations), and 10 repetitions were run for each combination (i.e., a total of 360 simulations was performed).

A one-way ANOVA of the simulation results was subsequently performed to determine the contribution of these parameters to the variability in the outcomes of the model (Ginovart et al., 2011). To do so, some representative outcomes of the model, namely time to 10^4^ bacilli (i.e., time to activation of immune response), Ebac at day 42, Ibac at day 42, Dbac at day 42, Kbac at day 42, percentage of infiltrated space at day 42, ratio between aAM and PMNs at day 42, the time at which PMNs exceeded aAM and the slope of aAM evolution at day 42, were chosen. Table 1 shows the *p*-value for each parameter and outcome considered. Table 2 broadens the one-way ANOVA for the three-level parameters, showing a multiple comparison analysis. In general, the lower the *p*-value, the higher the effect of that parameter on the corresponding outcome.

**Table 1:** **One-way ANOVA**.

		**Input parameters**			
		**AntiTh17**	**Encapsulation factor**	**Max-Ibac (BLTOL)**	**Drain-alveoli (BAM)**
Outcome variables	Time to 10^4^ bacilli	0.544	0.008^**^	<0.001^**^	<0.001^**^
	Ibac d42	<0.001^**^	0.005^**^	0.001^**^	0.024^*^
	Ebac d42	<0.001^**^	0.003^**^	<0.001^**^	<0.001^**^
	Dbac d42	<0.001^**^	0.003^**^	<0.001^**^	<0.001^**^
	Kbac d42	0.078	<0.001^**^	<0.001^**^	<0.001^**^
	% Infiltration d42	<0.001^**^	0.004^**^	<0.001^**^	0.002^**^
	Ratio aAM/PMNs d42	<0.001^**^	0.571	<0.001^**^	<0.001^**^
	Time-crossing aAM/PMNs	n/a	0.048^*^	<0.001^**^	<0.001^**^
	Slope aAM d42	0.058	0.434	<0.001^**^	0.006^**^

*P-values of simulation results when combining different input parameters and outcome variables (Welch's test was performed when the equality of variances requirement was not satisfied)*.

* *Statistically significant difference with p < 0.05*.

** *Statistically significant difference with p < 0.01*.

**Table 2:** **Multiple comparisons analysis**.

		**Input parameters**			
		**Max-Ibac (BLTOL)**		**Drain-alveoli (BAM)**	
Outcome variables	Time to 10^4^ bacilli	A-B-C		A-B-C	
		16–32	<0.001^**^	0.1–0.2	<0.001^**^
		32–64	<0.001^**^	0.2–0.3	<0.001^**^
		16–64	<0.001^**^	0.1–0.3	<0.001^**^
	Ibac d42	A-B-C		A-A-B	
		16–32	<0.001^**^	0.1–0.2	1.000
		32–64	0.009^**^	0.2–0.3	0.046^*^
		16–64	<0.001^**^	0.1–0.3	0.045^*^
	Ebac d42	A-A-B		A-B-B	
		16–32	0.993	0.1–0.2	<0.001^**^
		32–64	0.001^**^	0.2–0.3	0.673
		16–64	0.001^**^	0.1–0.3	<0.001^**^
	Dbac d42	A-A-B		A-A-B	
		16–32	0.426	0.1–0.2	0.990
		32–64	<0.001^**^	0.2–0.3	<0.001^**^
		16–64	<0.001^**^	0.1–0.3	<0.001^**^
	Kbac d42	A-B-C		A-B-C	
		16–32	<0.001^**^	0.1–0.2	<0.001^**^
		32–64	<0.001^**^	0.2–0.3	<0.001^**^
		16–64	<0.001^**^	0.1–0.3	<0.001^**^
	% Infiltration d42	A-B-B		A-B-B	
		16–32	<0.001^**^	0.1–0.2	0.005^**^
		32–64	0.065	0.2–0.3	0.998
		16–64	0.004^**^	0.1–0.3	0.006^**^
	Ratio aAM/PMNs d42	A-A-B		A-A-B	
		16–32	0.965	0.1–0.2	0.176
		32–64	<0.001^**^	0.2–0.3	<0.001^**^
		16–64	<0.001^**^	0.1–0.3	0.044^*^
	Time-crossing aAM/PMNs	A-B-C		A-B-A	
		16–32	<0.001^**^	0.1–0.2	<0.001^**^
		32–64	<0.001^**^	0.2–0.3	<0.001^**^
		16–64	<0.001^**^	0.1–0.3	0.145
	Slope aAM d42	A-B-C		AB-B-A	
		16–32	<0.001^**^	0.1–0.2	0.655
		32–64	<0.001^**^	0.2–0.3	0.011^*^
		16–64	<0.001^**^	0.1–0.3	0.070

*ANOVA for multiple comparisons with three-level parameters (Tukey method or Games-Howell method). Three different letters mean that there are no overlaps between intervals. The p-value for each pair is shown*.

* *Statistically significant difference with p < 0.05*.

** *Statistically significant difference with P < 0.01*.

## Results

### Bacillary load tolerability (BLTOL) of AM and breathing amplitude (BAM) determine time to immune response

**Figure 3:**
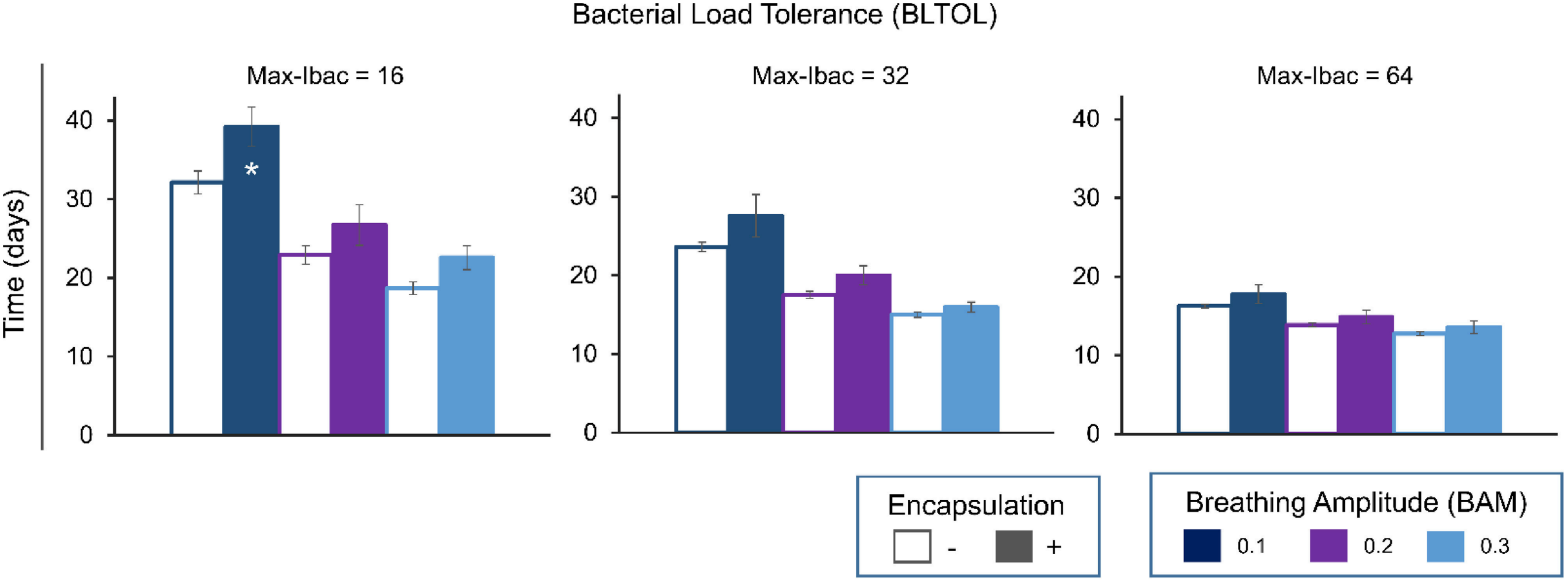
**Influence of maximum bacillary load per macrophage (Max-Ibac = 16, 32, 64) (BLTOL), encapsulation factor (−, no encapsulation, +, encapsulation) and breathing amplitude (BAM) (0.1, 0.2, 0.3) on the time to reach a total bacillary population of 10^4^ cells**. Results for AntiTh17 factor are not shown separately because no significant differences were found. Bars indicate the mean of 20 runs. ^*^Only 50% of runs reached the bacillary population of 10^4^ cells before day 42.

Figure 3 shows a clear negative correlation between BLTOL and BAM and the time to reach a total of 10^4^ bacilli. This threshold has been defined as the minimum bacillary load needed to trigger the immune response based on published experimental data (Jung et al., 2005; Vilaplana et al., 2014). The encapsulation process has a significant effect as regards increasing this period of time as it hampers bacillary dissemination.

### Breathing amplitude (BAM) is the most important factor influencing evolution of the bacillary load (BL)

**Figure 4:**
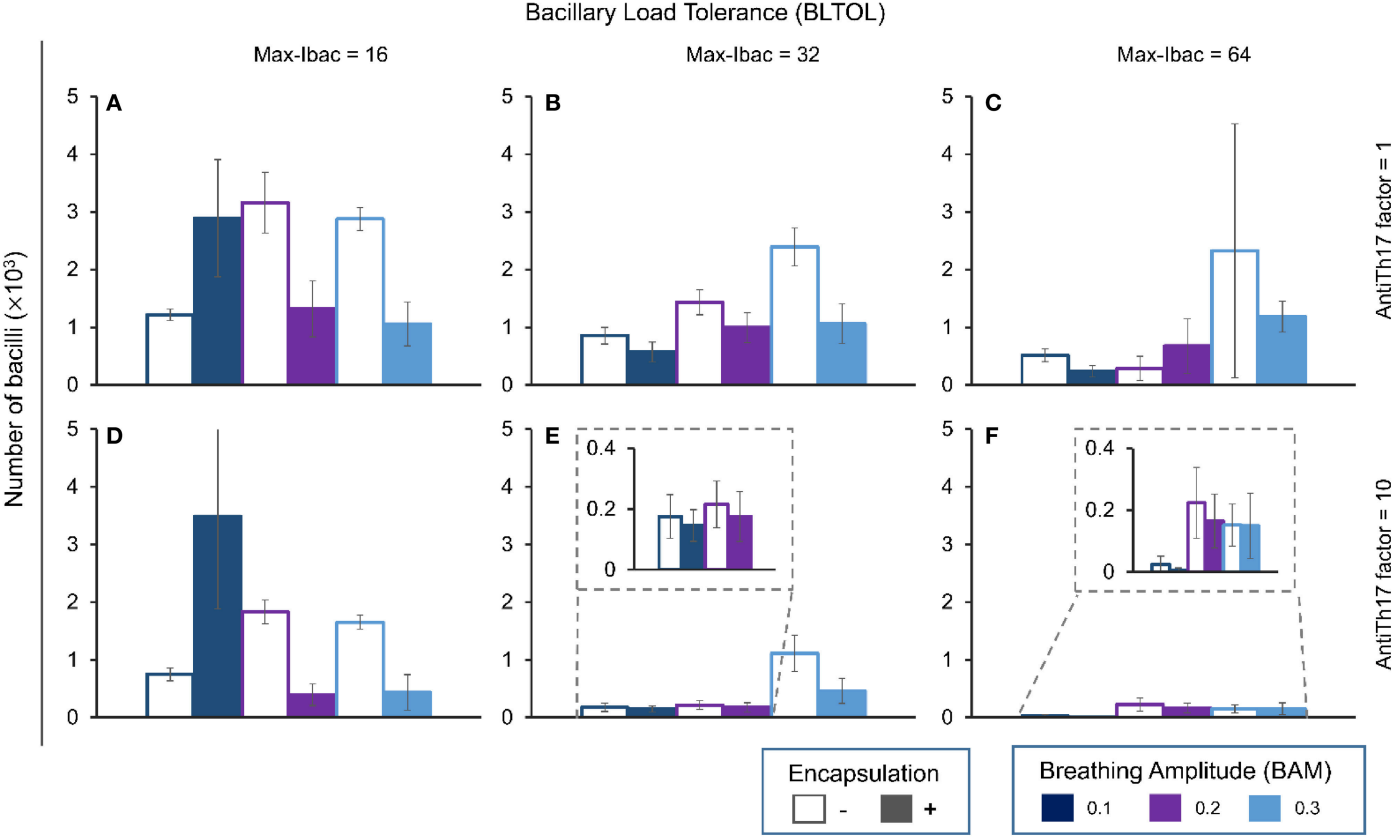
**Influence of different parameters on the number of intracellular bacilli at day 42**. Maximum bacillary load per macrophage (BLTOL): 16 **(A,D)**, 32 **(B,E)**, 64 **(C,F)**. AntiTh17 factor: 1 **(A–C)**, 10 **(D–F)** Encapsulation factor: −, no encapsulation, +, encapsulation. Breathing amplitude (BAM): 0.1, 0.2, 0.3. Bars indicate the mean of 10 runs.

**Figure 5:**
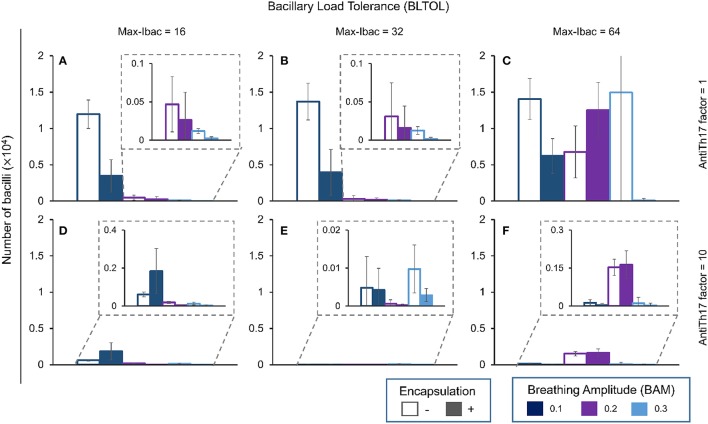
**Influence of different parameters on the number of extracellular bacilli at day 42**. Maximum bacillary load per macrophage (BLTOL): 16 **(A,D)**, 32 **(B,E)**, 64 **(C,F)**. AntiTh17 factor: 1 **(A–C)**, 10 **(D–F)** Encapsulation factor: −, no encapsulation, +, encapsulation. Breathing amplitude (BAM): 0.1, 0.2, 0.3. Bars indicate the mean of 10 runs.

**Figure 6:**
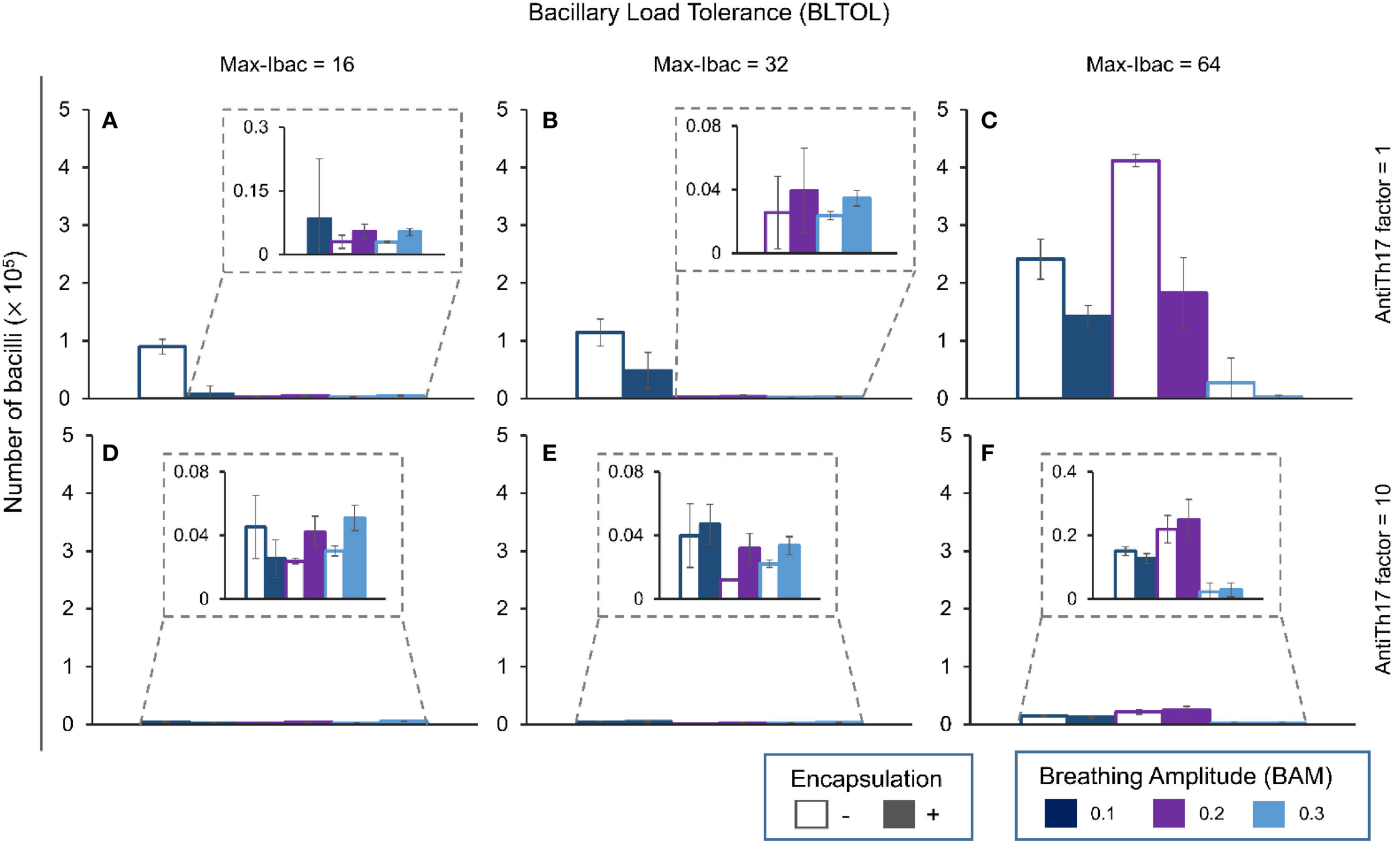
**Influence of different parameters on the number of dormant bacilli at day 42**. Maximum bacillary load per macrophage (BLTOL): 16 **(A,D)**, 32 **(B,E)**, 64 **(C,F)**. AntiTh17 factor: 1 **(A–C)**, 10 **(D–F)** Encapsulation factor: −, no encapsulation, +, encapsulation. Breathing amplitude (BAM): 0.1, 0.2, 0.3. Bars indicate the mean of 10 runs.

**Figure 7:**
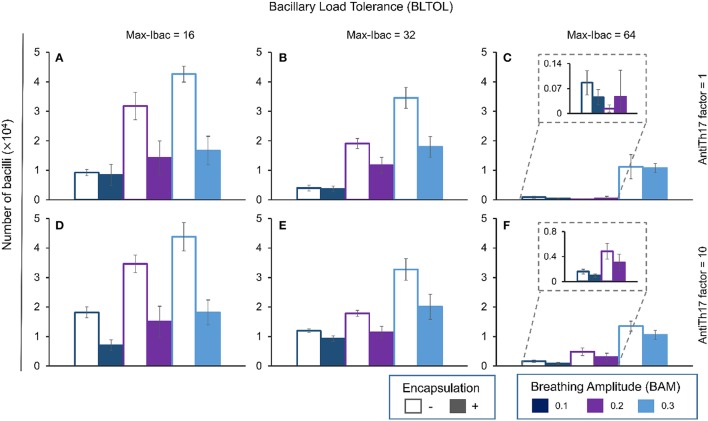
**Influence of different parameters on the number of killed bacilli at day 42**. Maximum bacillary load per macrophage (BLTOL): 16 **(A,D)**, 32 **(B,E)**, 64 **(C,F)**. AntiTh17 factor: 1 **(A–C)**, 10 **(D–F)** Encapsulation factor: − = no encapsulation, + = encapsulation. Breathing amplitude (BAM): 0.1, 0.2, 0.3. Bars indicate the mean of 10 runs.

Figures 4–**7** show the BL for the four bacillary forms considered at day 42. The intracellular bacilli (Ibac) analysis (Figure 4) shows a positive correlation with BAM, with one exception in the case of a low BLTOL value, where the influence of BAM reaches a plateau at the value of 0.2.

Encapsulation helps control the BL with one exception: it favors an increase in bacillary load at an amplitude of 0.1. Curiously, the antiTh17 factor also exhibits the same behavior. These findings are logical as an increase in BAM facilitates dissemination of the bacillary load, thus increasing expansion and the possibility for intracellular growth. In addition, the anti-Th17 response can be explained by its ability to stop PMN infiltration and promote the entry of aAMs.

In contrast to what happens with the Ibac, there is a negative correlation between evolution of the Ebac concentration and the BAM (Figure 5). Again, this is logical as the accumulation of bacilli favors the attraction of PMNs. This pattern changes at the highest BLTOL, where there is a wide variation. This can be explained by the fact that, once this value is reached (Max-Ibac = 64), PMNs are immediately attracted, thus meaning that BAM has only a limited influence. Both behaviors are reflected in Table 2, where an increase in BAM to 0.3 does not make any difference, whereas an increase in BLTOL to Max-Ibac = 64 induces significant changes.

The role of encapsulation tends to prevent Ebac progression, although there are multiple exceptions. One explanation could be that the speed of infiltration is greater than the ability to encapsulate. What is more understandable is the role of anti-Th17, which stops Ebac progression in all circumstances by abrogating PMN infiltration.

The pattern for Ebac is almost the same as that for Dbac (Figure 6), although in this case the highest BAM value appears to have an influence on the reduction in its concentration in the context of the highest BLTOL.

Finally, Kbac (Figure 7) seems to evolve in a similar manner to Ibac as it is positively correlated with BAM. In this case, the higher the BLTOL the lower the Kbac. Increasing BLTOL promotes neutrophilic infiltration, and the fate of these bacilli is to become dormant (Dbac) in the context of necrotic tissue rather than being killed. As such, the results are coherent. In this case, the encapsulation process clearly reduces the Kbac concentration. This can be explained by control of the dissemination, which results in an overall lack of living bacilli, and thus also necrosed bacilli. What is more intriguing is the lack of influence of the antiTh17 response, as this should stop PMN infiltration and favor bacillary necrosis.

### The infiltration percentage and quality of the lesion are strongly influenced by the breathing amplitude (BAM)

We define four types of lesions according to two different readouts, i.e., their quality and the infiltration percentage (Table 3):

A.- Proliferative in progression, with low numbers of Dbac and a high proportion of Kbac (**Figure 10**, pictures B, C, F, b, c, and f) showing a high granulomatous infiltration.
B.- Proliferative controlled, as there is a very low number of Dbac and a very high proportion of Kbac showing a low granulomatous infiltration.
C.- Exudative, based on a large necrotic center surrounded by an active ring of alveoli filled with PMNs and massive presence of Dbac (**Figure 10** pictures A,G,J,M,N,P, and Q) showing a high granulomatous infiltration.
D.- Exudative controlled, with smaller lesions than in type C surrounded by a ring of aAMs (**Figure 10** pictures g, j, m, p, n, q, and r).

**Table 3:** **Phenotypical characteristics of the lesions according to the four main factors previously identified as being able to favor the infiltration of *Mtb*-infected lesions with PMNs**.

	**Max-Ibac (BLTOL)**								
		**16**		**32**		**64**			
Breathing Amplitude (BAM)	0.1	**C**	**C**	**C**	**C**	**C**	**C**	1	Anti-Th17
	0.2	**A**	**B**	**B**	**B**	**C**	**C**		
	0.3	**A**	**A**	**B**	**B**	**B**	**B**		
	0.1	**B**	**B**	**D**	**D**	**D**	**D**	10	
	0.2	**A**	**B**	**B**	**B**	**D**	**D**		
	0.3	**A**	**A**	**B**	**B**	**B**	**D**		
		−	+	−	+	−	+		
	Encapsulation								

*These are the tolerability of infected macrophages to the bacillary load (BLTOL); the capacity to modulate the Th17 response; the breathing amplitude (BAM); and the encapsulation of Mtb-infected lesions. These results are obtained after running the program until day 46 post-challenge. **Figure 10** shows the phenotype. **Type A**.- In dark blue, proliferative in progression, with low quantities of Dbac and a high proportion of Kbac. **Type C**.- In red, exudative, based on a large necrotic center surrounded by an active ring of alveoli filled with PMNs and the massive presence of Dbac. **Type D**.- In orange, exudative controlled, with smaller lesions than in type C surrounded by a ring of aAMs. **Type B**.- In sky blue, proliferative controlled, as there is a very low quantity of Dbac and a very high proportion of Kbac*.

**Figure 8:**
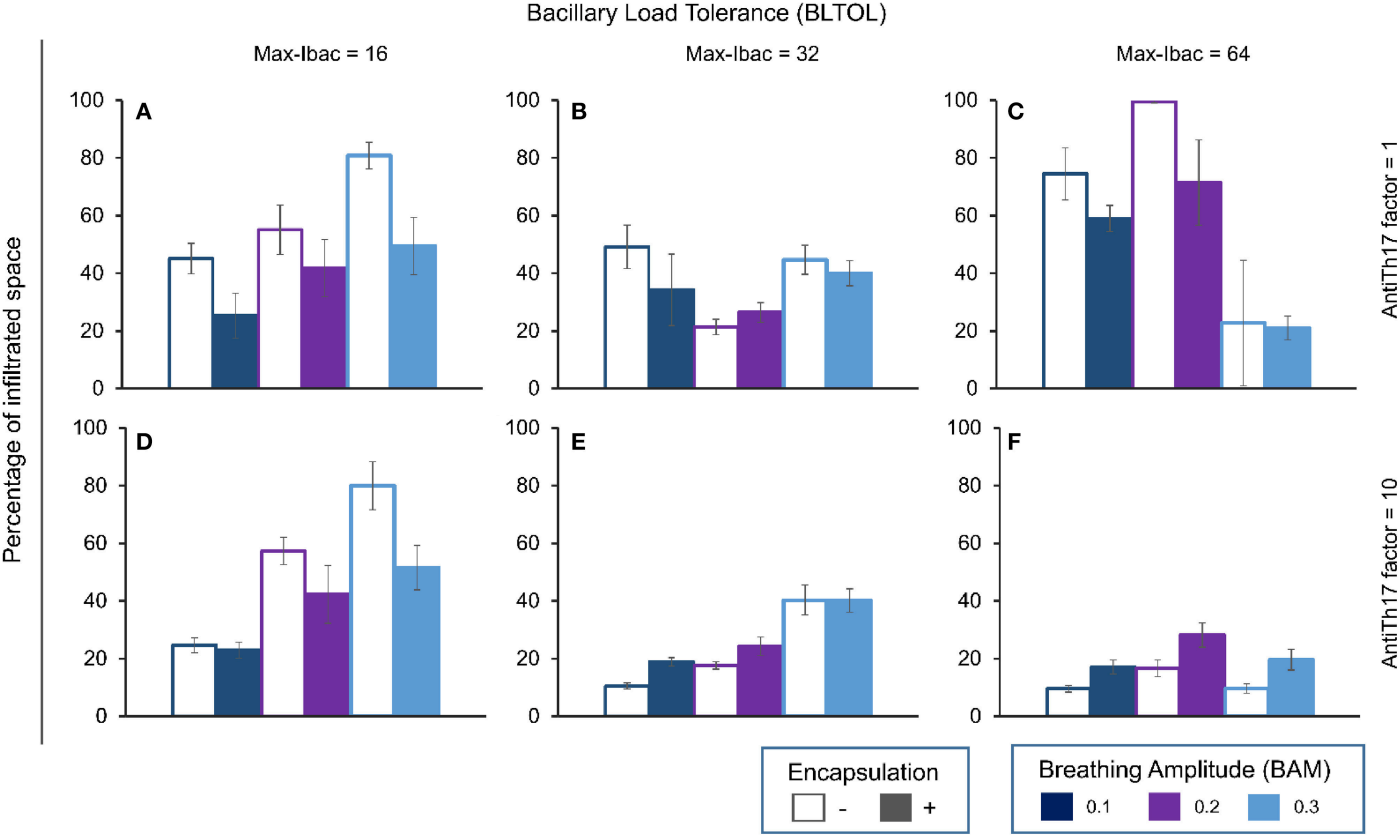
**Influence of different parameters on the percentage of infiltrated space at day 42**. Maximum bacillary load per macrophage (BLTOL): 16 **(A,D)**, 32 **(B,E)**, 64 **(C,F)**. AntiTh17 factor: 1 **(A–C)**, 10 **(D–F)** Encapsulation factor: − = no encapsulation, + = encapsulation. Breathing amplitude (BAM): 0.1, 0.2, 0.3. Bars indicate the mean of 10 runs.

**Figure 9:**
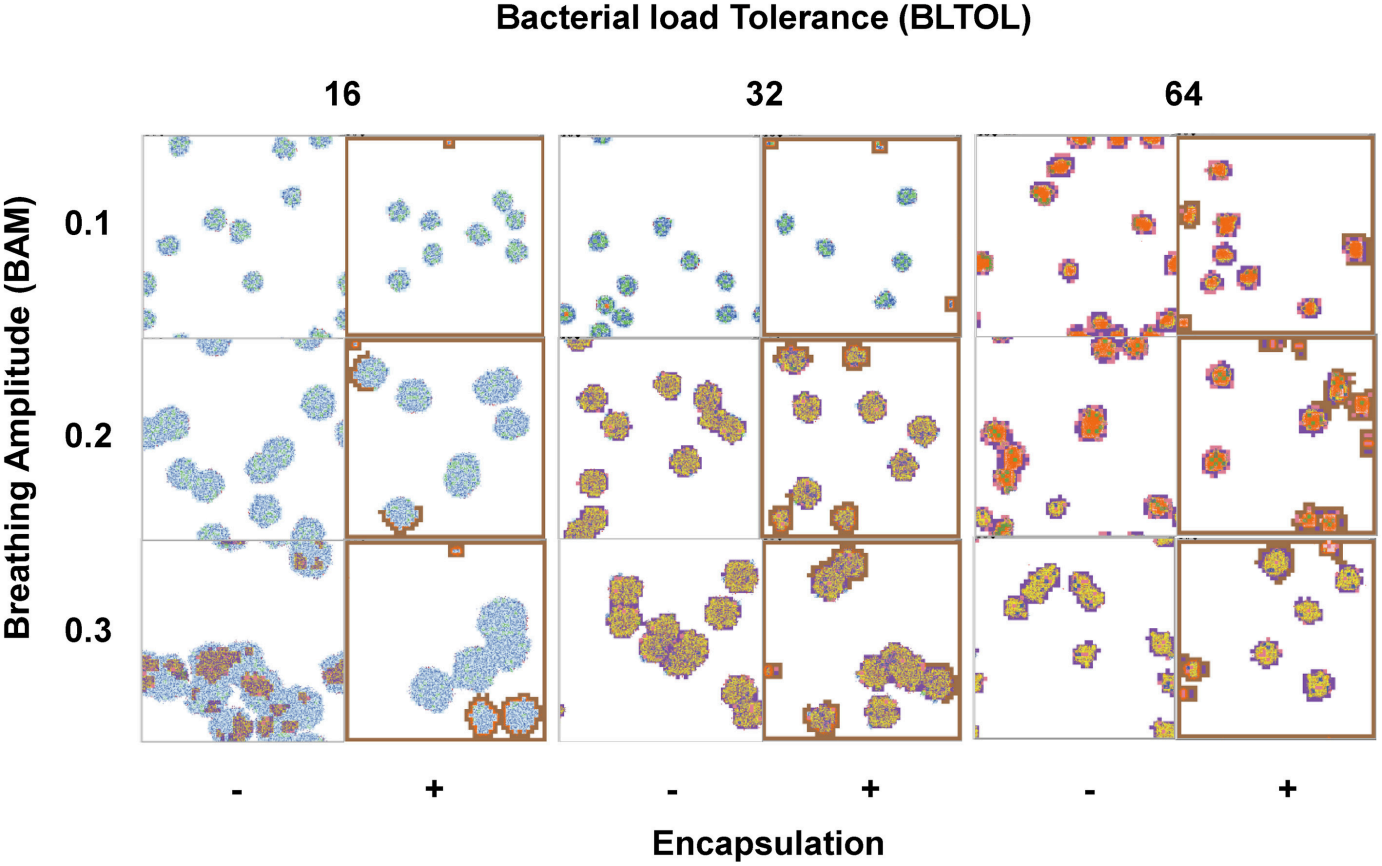
**Phenotypical characteristics of the lesions after running the program until day 21 post-challenge**. **Colors**: Alveoli: **White**: when a single AM is present; **Black**: when the AM is destroyed; **Blue**: when the AM is destroyed and replaced by another AM from the interstitium; **Violet**: when an activated AM (aAM) is present; **Pink**: when PMNs occupy the alveoli; **Brown**: capsule. Bacilli: **Blue** challenge bacilli; **Red**: Intracellular bacilli (Ibac); **Green**: Extracellular (Ebac); **Brown**: growing Ebac; **Orange**: dormant (Dbac); **Yellow**: killed bacilli (Kbac).

**Figure 10:**
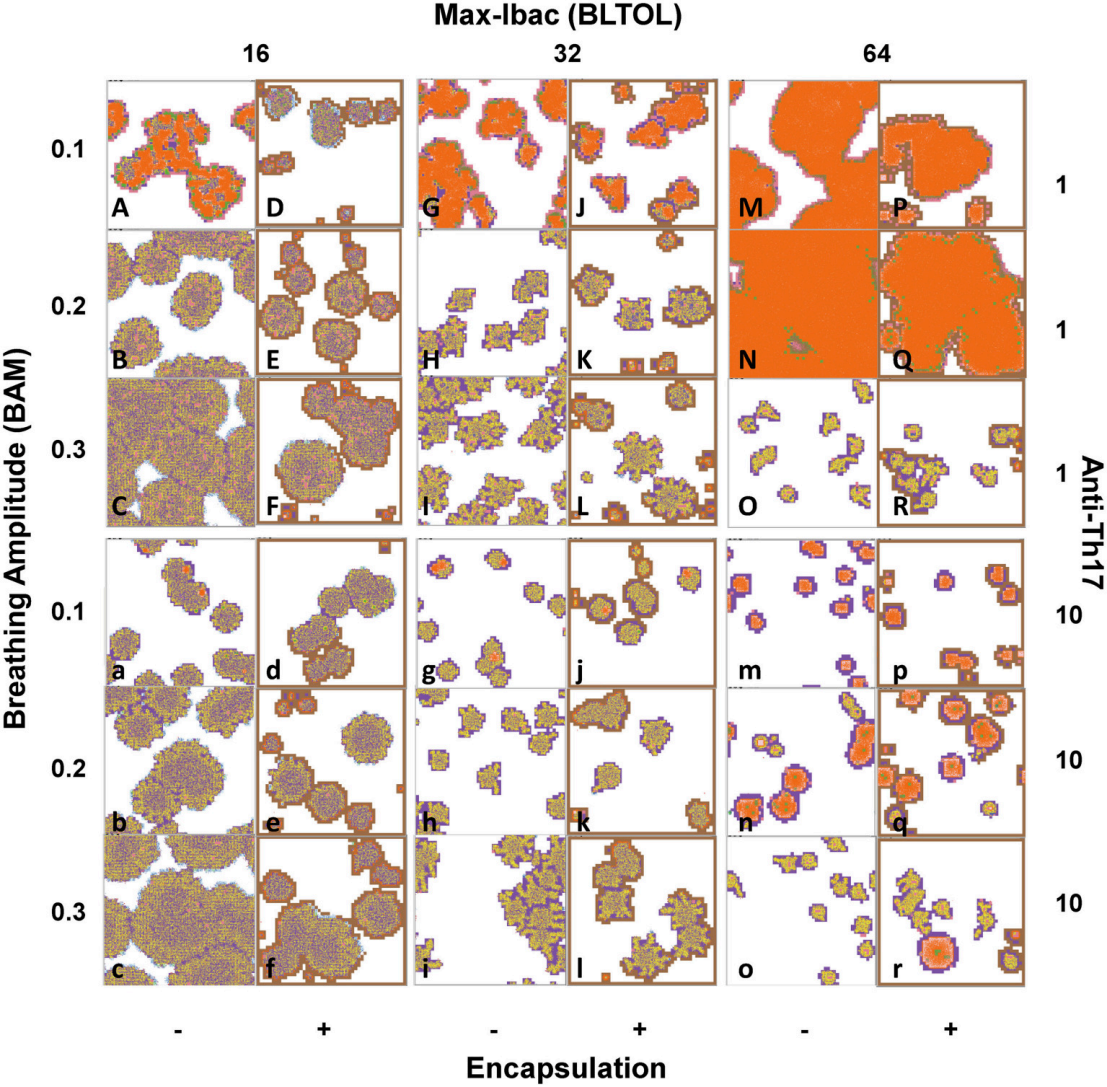
**Phenotypical characteristics of the lesions after running the program until day 46 post-challenge**. **Type A**.- Proliferative in progression, with low quantities of Dbac and a high proportion of Kbac (pictures B, C, F, b, c and f). **Type C**.- Exudative, based on a large necrotic center surrounded by an active ring of alveoli filled with PMNs and the massive presence of Dbac (pictures A, G, J, M, N, P and Q). **Type D**.- Exudative controlled, with smaller lesions than in type C surrounded by a ring of aAMs (pictures g, j, m, p, n, q and r). **Type B**.- Proliferative controlled, as there is a very low quantity of Dbac and a very high proportion of Kbac (all other pictures). ***Colors:*** Alveoli: **White**: when a single AM is present; **Black**: when the AM is destroyed; **Blue**: when the AM is destroyed and replaced by another AM from the interstitium; **Violet**: when an activated AM (aAM) is present; **Pink**: when neutrophils occupy the alveoli; **Brown**: capsule. Bacilli: **Blue** challenge bacilli; **Red**: Intracellular bacilli (Ibac); **Green**: Extracellular (Ebac); **Brown**: growing Ebac; **Orange**: dormant (Dbac); **Yellow**: killed bacilli (Kbac).

At low BLTOL, BAM is positively correlated with the percentage infiltration, and encapsulation clearly decreases it, whereas the anti-Th17 response only has an effect at low BAM (Figure 8). As can be seen from Figure 9 together with Figure 4, there is no immune response at day 21 in the majority of the cases. Figure 10 shows the progression at day 46, with infiltration increasing with an increase in BAM. Looking at the quality of the lesion, it can be seen that the lesions are mainly based on AM and Kbac, thus suggesting type B lesions that become type A at high BAM. There is only one case in which type C lesions can be seen, with low BAM, no encapsulation, and a massive presence of Dbac being detected (see Figure 6).

An unusual profile is observed at medium BLTOL, with both low and high BAM inducing higher infiltration, although this can be decreased with encapsulation (Figure 8). In this case, the antiTh17 response is able to reduce infiltration at low BAM. Looking at the appearance of the lesions on d21 (Figure 9), there appears to be a slight increase in infiltration with BAM, which is also reflected in the delay in the immune response at low BAM. In contrast, the appearance of the lesions is very similar at day 46 (Figure 10) in terms of both infiltration and quality (type B lesions), thus reflecting the presence of activated AM (aAM) with Kbac. However, in the case of low BAM, the lesions are mainly type C.

At high BLTOL, infiltration increases with an increase in BAM from 0.1 to 0.2, and, logically, anti-Th17 has a critical influence on controlling this infiltration as it is mainly caused by the accumulation of PMNs and extracellular growth (Figure 8). This can clearly be seen from Figure 9, which shows the necrosis caused by the accumulation of PMNs and the presence of Dbac. The fact that this becomes marked by day 46 is interesting as the immune response appears at almost the same time (Figure 3) for all three BAM values. The lesions for the lower and medium BAM values are of type C and are therefore difficult to control by encapsulation. In this case, as can be seen from Figure 8, anti-Th17 clearly transforms these lesions to type D. Interestingly, the highest BAM also induces a radical change in the fate of the lesions, from exudative to proliferative, which clearly become type B.

**Figure 11:**
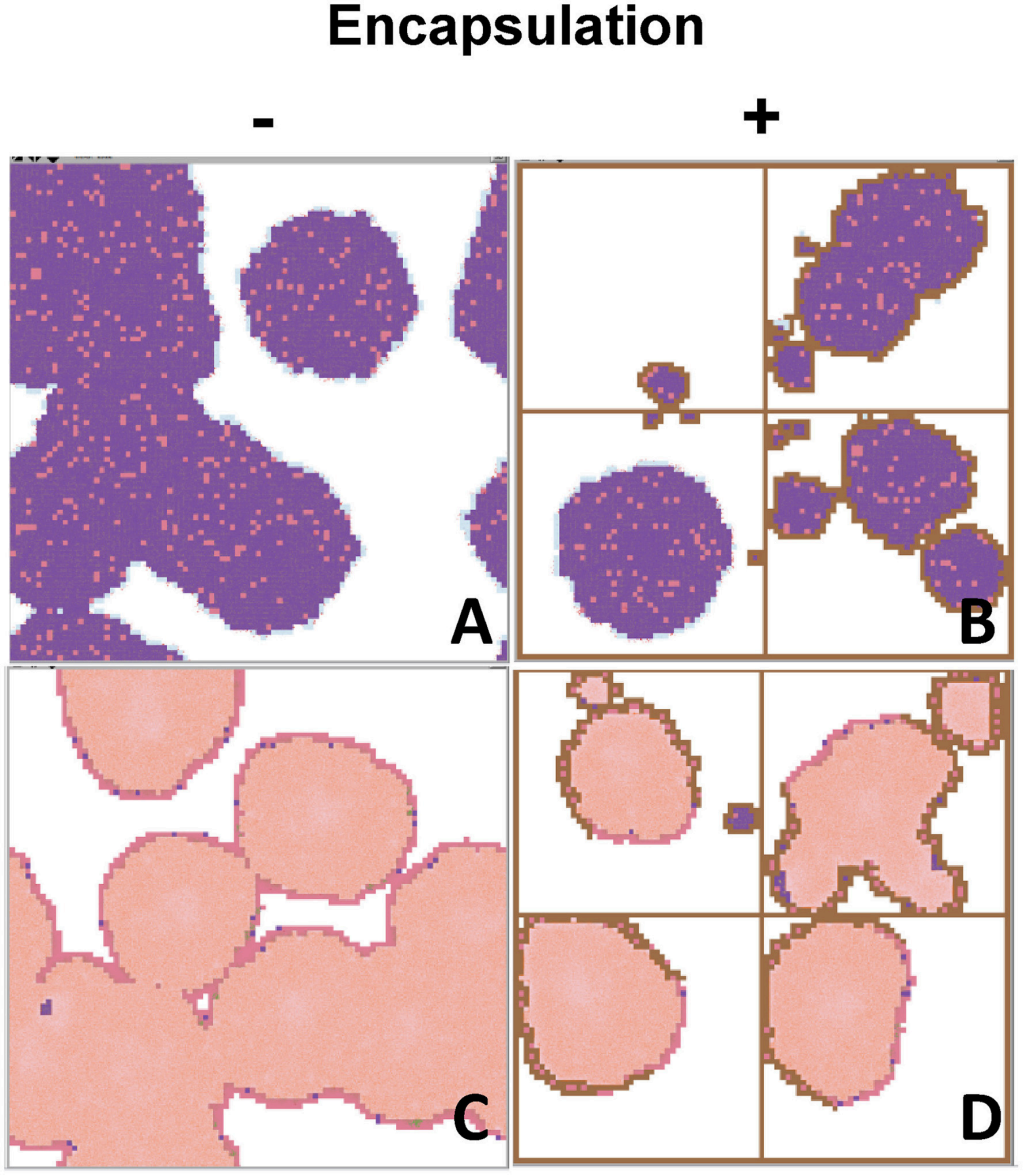
**Interface of the program using four-times more patches to show how important the encapsulation process is in a wider grid as regards stopping the progression toward active TB**. This is the case of a typical proliferative response, with low BLTOL and high BAM **(A,B)**, and a medium BLTOL and low BAM **(C,D)**, after running the program until day 46 post-challenge. ***Colors:*** Alveoli: **White**: when a single AM is present; **Black**: when the AM is destroyed; **Blue**: when the AM is destroyed and replaced by another AM from the interstitium; **Violet**: when an activated AM (aAM) is present; **Pink**: when neutrophils occupy the alveoli; **Brown**: capsule. Bacilli: **Blue** challenge bacilli; **Red**: Intracellular bacilli (Ibac); **Green**: Extracellular (Ebac); **Brown**: growing Ebac; **Orange**: dormant (Dbac); **Yellow**: killed bacilli (Kbac).

Figure 11 is the result of increasing the size of the canvas four-fold, thus representing four secondary lobes instead of just one, in order to gain a better understanding of the importance of the encapsulation factor in stopping progression of the lesions, both proliferative and exudative.

Overall, even though it originally seems that BLTOL is the most important factor for lesions to become exudative, a proposal supported by the global statistical analysis (Table 2), which seems to show that BLTOL is slightly more influential than BAM, it is clear that, qualitatively speaking, exudative lesions are always induced at low BAM but the encapsulation process can abrogate this at low BLTOL.

### The slope in aAM evolution and in the aAM/PMN ratio also defines the degree of infiltration and the quality of the lesions

**Figure 12:**
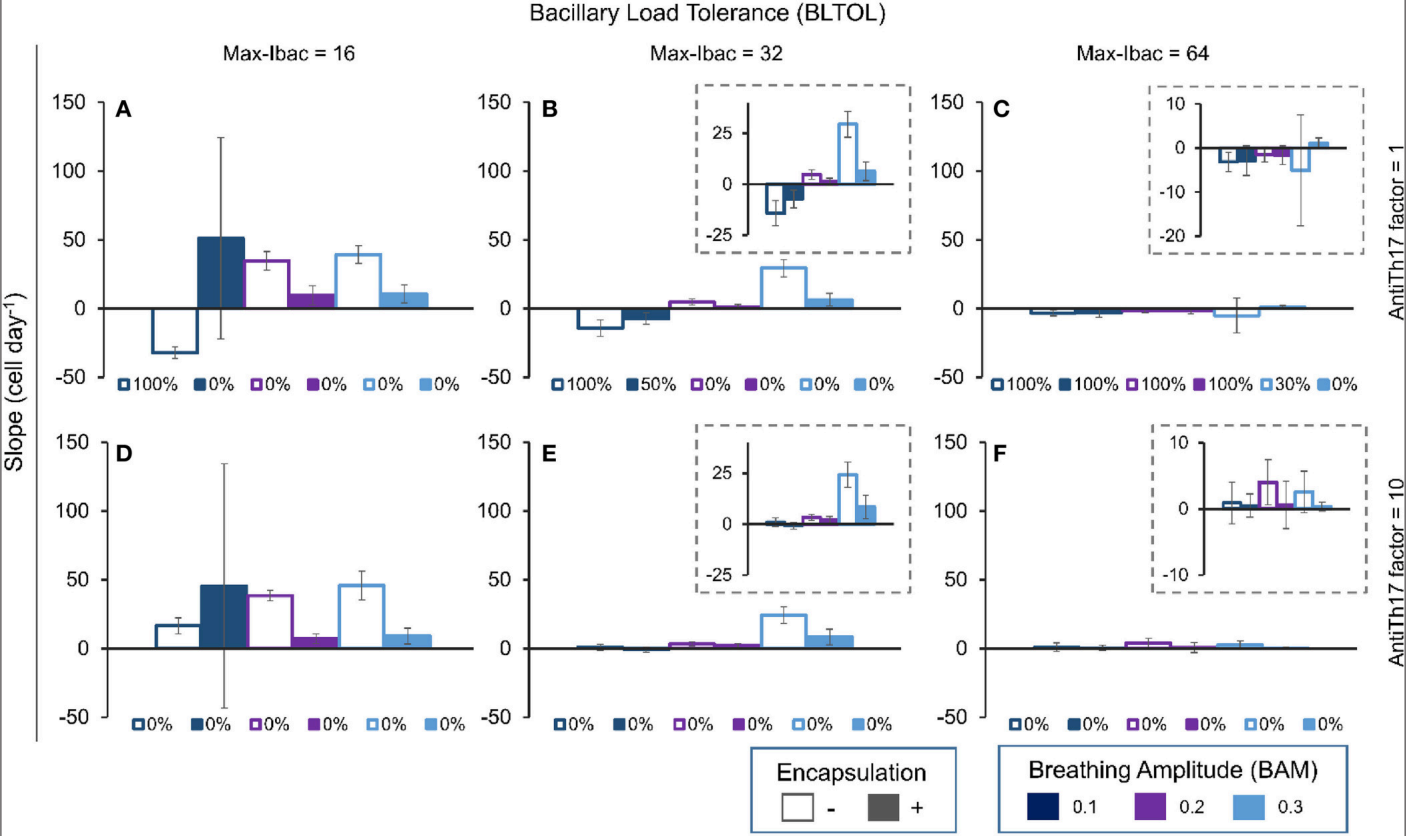
**Influence of different parameters on the slope of aAM evolution at day 42**. Maximum bacillary load per macrophage (BLTOL): 16 **(A,D)**, 32 **(B,E)**, 64 **(C,F)**. AntiTh17 factor: 1 **(A–C)**, 10 **(D–F)** Encapsulation factor: −, no encapsulation, +, encapsulation. Breathing amplitude (BAM): 0.1, 0.2, 0.3. Bars indicate the mean of 10 runs. Individual legends indicate the percentage of 10 runs in which the PMN population exceeded the aAM population.

**Figure 13:**
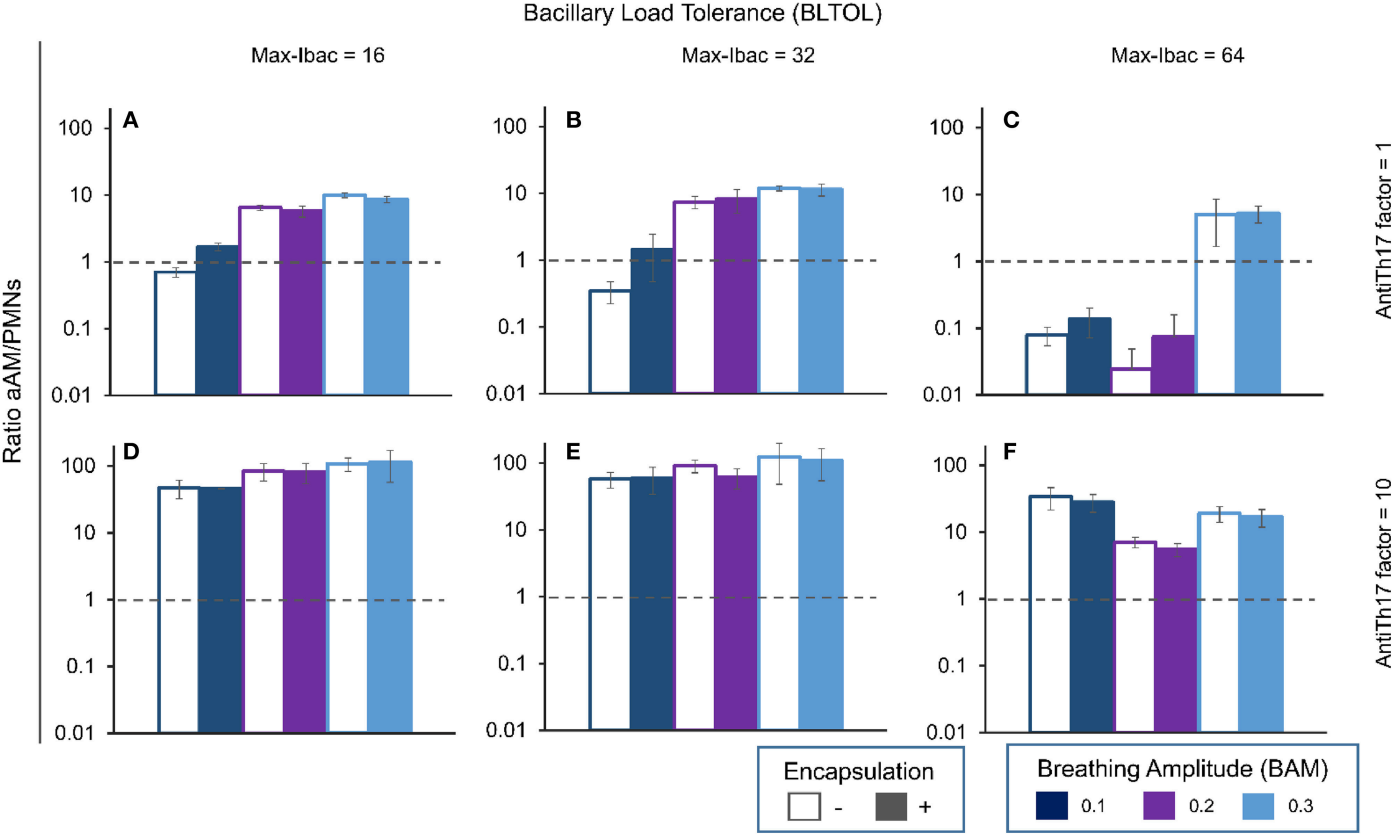
**Influence of different parameters on the ratio between aAMs and PMNs at day 42**. Maximum bacillary load per macrophage (BLTOL): 16 **(A,D)**, 32 **(B,E)**, 64 **(C,F)**. AntiTh17 factor: 1 **(A–C)**, 10 **(D–F)** Encapsulation factor: − = no encapsulation, + = encapsulation. Breathing amplitude (BAM): 0.1, 0.2, 0.3. Bars indicate the mean of 10 runs. Horizontal dotted lines correspond to ratio 1 (ratio > 1 corresponds to aAM > PMNs, and ratio <1 corresponds to aAM < PMNs).

It can be seen from Figure 12, which shows the slope in aAM evolution at day 42, that the higher the slope the higher the infiltration, but in this case the value is negative as neutrophilic infiltration predominates. This can also be seen when the aAM/PMN ratio is less than 1 (Figure 13).

The aAM/PMN ratio reflects the quality of the lesions (Figure 13). A ratio >1 defines the dominance of aAMs in the lesions, and thus the presence of a proliferative-type activated macrophage lesion. Exudative lesions can be detected when this ratio is <1. Interestingly, an increase in anti-Th17 clearly reverts the exudative lesions to proliferative (values increasing from <1 to >1).

## Discussion

The modeling of biological systems allows us to determine and evaluate the effect of different factors in an environment that would be very difficult to predict or even to experiment using *in vitro* or *in vivo* tools. So far a lot of efforts have been already done to understand the granuloma induction in TB infection, specially by the group of Kirschner (Segovia-Juarez et al., 2004; Marino et al., 2011; Gideon et al., 2015). Those works are characterized by the use of a multiscale process that has more emphasis on the molecular and cellular scale together with the tissue and organ scale, including from the antigen presentation phenomenon, cytokine/chemokine diffusion dynamics, or the trafficking between the lung and the draining lymph node. Their works usually combine the use of IBM and partial differential equations for continuous variables. The originality of our proposal is that we introduce a combination of two scales. In particular our data help us to understand the role of four factors related to the neutrophilic infiltration of Mtb lesions, and thus to the induction of TB. Two of these, namely the BAM and the encapsulation capacity of the intralobar septae, are anatomical. These two factors have been combined with another two that facilitate neutrophilic infiltration, which could be related to the quality of the immune response and, to some extent, to some degree of genetic susceptibility (Tobin et al., 2012). These are the tolerability of the intracellular bacillary load (BLTOL) (Ayres and Schneider, 2008) and the ability to counterbalance the Th17 response during the immune phase. Although BLTOL has received relatively little attention, it could be related to a known defensive mechanism against intracellular pathogens, namely apoptosis or organized cell-death, rather than necrosis, which is unregulated (Dey and Bishai, 2014). In this case we have related BLTOL to the ability of AM to hold more or less bacilli and, as has been demonstrated experimentally, this holding capacity (or tolerability) with the ability to attract PMNs. This is because *in vitro* experiments have demonstrated that a high proportion of Mtb bacilli per macrophage, known as multiplicity of infection (MOI), induces more necrosis (Lee et al., 2006), which in turn is related to a greater attraction of PMNs (Gan et al., 2008). We have defined a parameter to represent the way in which PMNs are attracted once a certain number of extracellular bacilli at an alveolar site has been reached (*Ebacs-PMN*) simply because the maximum MOI has been reached.

Similarly, the quality of the immune response has been linked to the ratio between extracellular and extracellular-plus-intracellular bacilli. As the Th17 response is associated with extracellular pathogens (Korn et al., 2009), this proportion has been found to be the most easily correct to determine this response understood as the capacity to attract PMNs. What could this so-called anti-Th17 factor be? Again, this is a virtual concept that could include anything from a genetic factor to a new prophylactic-therapeutic approach that can trigger it.

BAM has been associated with bacillary drainage capacity. The values of this drainage capacity are scaled to the capacity to drain the bacilli to neighboring alveoli as a result of the breathing activity. This concept attempts to take into account the anatomical particularities of the lung parenchyma, which are organized into alveolar sacs that, in turn, are linked via the bronchiole to form acini (Webb, 2006).

Although there are fibroblasts in the entire parenchyma, we assumed that the encapsulation of granulomas is driven by fibroblasts of interlobular septae. This is in accord with observations by Gil et al. (2010). The encapsulation process has been defined by assigning a 100% likelihood of being generated if a normal alveolus has a capsule and another occupied alveoli (by PMN or activated macs) as its neighbor. This gives very reliable values when compared with the minipig model (Gil et al., 2010) in terms of the size of encapsulated lesions, which can be as small as 0.5 mm. A very interesting proposal in this regard is that the high expansion speed of the exudative lesions overwhelms the encapsulation process. This image has traditionally been interpreted as the ability of a growing lesion to destroy the capsule (Grosset, 2003). However, this is a very difficult process. Indeed, in a similar process, namely necrotizing fasciitis, it is very difficult to destroy the fascia that encapsulates the muscles, thus resulting in necrosis of the muscle (Henningham et al., 2015). We are faced with a very similar scenario here in which the most important aspect is the ability to be faster than encapsulation. If this is the case, then there is a chance to overcome it.

It is interesting to note that the presence of PMNs in exudative lesions is limited to just the outer ring, as we have also seen in the C3HeB/FeJ murine model (Marzo et al., 2014) and in human lesions (Cardona, 2015), and that the necrotic center contains large numbers of dormant bacilli trapped in necrotic tissue. Although these are not usually seen using conventional stains (i.e., acid fast stain like Ziehl Neelsen's), the use of specific antibodies has demonstrated the massive presence of bacilli in necrotic tissue (Seiler et al., 2003).

The capacity to drain bacilli (BAM), clearly an anatomical factor, is very important as regards controlling the size and quality of the lesions. Thus, higher BAM values increase the possibility of infecting neighboring AMs, which results in faster bacillary growth and thus a quicker induction of the immune response. Similarly, a high BAM also allows for constant drainage and a lower local accumulation of bacilli, which eventually stops the infiltration of PMNs. This is why one interesting finding of this study is that the highest BLTOL induces a higher number of progressive exudative lesions, which is suddenly stopped when a high BAM is reached. This model therefore shows the importance of BAM in the induction of exudative lesions. In the majority of cases, a low value of BAM has been linked to induction of this type of lesion, and only in one case, with a low bacillary tolerability (BLTOL), is the encapsulation process able to stop this.

The second most important factor with regard to the quality of lesions is the degree of tolerability to intracellular bacilli (BLTOL), a factor that is probably defined genetically (Tobin et al., 2012). This is logical as it is decisive for determining local infiltration by PMNs. As regards the values chosen, and considering previous experimental data, the value Max-Ibac = 16 is probably the least realistic one for humans, whereas the other two appear to be more normal in light of the experimental data reported by Lee et al. (2006), where the intracellular bacillary load was usually between 40 and 60 bacilli. Interestingly, this value could be suitable for the mouse infection as the size of the AM is, on average, half that in humans (Suarez et al., 2012). This is interesting as mice clearly develop such progressive proliferative lesions whereas the proliferative lesions developed in humans are not progressive (Cardona, 2015).

Our findings also show how an anti-Th17 response could be particularly useful for stopping progression of the lesions, except in the case of low tolerability (BLTOL), where the presence of PMNs is very low, thus showing that in this case the Th17 response would not be very important. As this low BLTOL could be related to previous findings in mice, this could explain why an anti-Th17 factor had only a minimal influence in defining new experimental options against TB.

Finally, the model shows the importance of the encapsulation process as regards stopping the progression of lesions, and also how this protective factor can be overwhelmed, especially by progressive exudative lesions, which are the ones usually related to the induction of cavities (Cardona, 2015). Further work should include a 3D spatial structure of the lung with septae in order to better account for the encapsulation process. In addition, such further work should also include a mode in-depth uncertainty and sensitivity analysis that was able to capture the tiny dynamics of the model from a global perspective (Marino et al., 2008), which was not tackled in this paper.

Overall, this model has related four factors that would be impossible to relate in a single experimental model and, perhaps more importantly, as the interface is friendly, any interested person can modify and interact with this model to better understand or even better describe the process of evolution from infection to active TB. The simulator TBPatch.nlogo can be downloaded from http://mosimbio.upc.edu/ or https://unitatdetuberculosiexperimental.wordpress.com/.

## Author contributions

PC conceived, designed and implemented the model. CP supervised the model's design and implementation. Both designed and carried out *in silico* experiments, critically analyzed and discussed the results and wrote the manuscript.

### Conflict of interest statement

The authors declare that the research was conducted in the absence of any commercial or financial relationships that could be construed as a potential conflict of interest.
