# Supplementary material for: The Small Breathing Amplitude at the Upper Lobes Favors the Attraction of Polymorphonuclear Neutrophils to Mycobacterium tuberculosis Lesions and Helps to Understand the Evolution toward Active Disease in An Individual-Based Model
Source: Front Microbiol. 2016 Mar 29;7:354. doi: 10.3389/fmicb.2016.00354 (PMC4810076; doi:10.3389/fmicb.2016.00354)
Supplement: Supplementary file 1 [file Table1.PDF]

Table S1: Details of the parameters involved in the model together with their values and stage where these were fixed. (PE=Parameter Estimation)

Parameter	Fixed/Explored	Value(s)	Comments or details
Doubling-time	Fixed a priori	24 hours	This biological value was taken from (North 2004)
Bacs-immunity	Fixed a priori	10000 bacillli	From bibliography (Vilaplana et al. 2014)
Initial-bac	Fixed a priori	10 bacilli	Related with initial conditions. We fixed it to 10, in order to homogenize initial conditions of all simulations. It was an arbitrary choice. Further work could explore lower and higher values.
Reinfect	Fixed at PE stage	5×10^{-4} %	A very low value was chosen, close to 0
Ebacs-PMN	Fixed at PE stage	64 bacilli / patch	We chose this number as this is the number of the maximum tolerance of one infected macrophage.
Immunity-Kbac	Fixed at PE stage	30 %	This value is in the range of the killing activity of infected macrophages *"in vitro"* (Lee et al. 2006)
Inflamm-attract	Fixed at PE stage	75 bacilli	It was fixed as a half of the *Max-harbour* capacity of the alveoli. Thus with enough inflammatory response to attract the lymphocytes.
Max-harbour	Fixed at PE stage	150 bacilli/patch	It was fixed to have a magnitude of around the maximum bacillary load tolerable by two macrophages.
Drain-tissue	Fixed at PE stage	Drain-alveoli /2	The drainage distance through tissue was considered to be the half of the local rate through the bronchial tree.
Drain-Dbac	Fixed at PE stage	Drain-alveoli*10	The drainage distance through foamy macrophage was assumed to be one order of magnitude higher than local drainage.
Max-Ibac	Delimited at PE stage and selected for being explored	16, 32 and 64 bacilli	Explored values fixed according to bibliography (Lee et al. 2006)
Drain-alveoli	Delimited at PE stage and selected for being explored	0.1, 0.2 and 0.3 patch h^{-1}	There is a factor 3 between BAM at the lower lobes and upper lobes (Guo, Xu, and Shi 2011). This factor was established between extreme values considered.
Anti-Th17 factor	Delimited at PE stage and selected for being explored	1 and 10	Adimensional factor. The explored values include no presence of this factor (*Anti-Th17 factor=1*) and an anti-Th17 presence (*Anti-Th17 factor=10*)
Encapsulation-ratio	Delimited at PE stage and selected for being explored	0 and 1	Adimensional probability. Explored values inlcude no encapsulation (*Encapsulation-ratio=0*) and encapsulation (*Encapsulation-ratio=1*). Intermediate values would mean slow encapsulation rate, it has not been explored in this paper.
